# Underwater Wavelength Attack on Discrete Modulated Continuous-Variable Quantum Key Distribution

**DOI:** 10.3390/e26060515

**Published:** 2024-06-14

**Authors:** Kangyi Feng, Yijun Wang, Yin Li, Yuang Wang, Zhiyue Zuo, Ying Guo

**Affiliations:** 1 School of Automation, Central South University, Changsha 410083, China; 2 School of Computer Science, Beijing University of Posts and Telecommunications, Beijing 100876, China

**Keywords:** wavelength attack, continuous-variable quantum key distribution, discrete modulated, underwater

## Abstract

The wavelength attack utilizes the dependence of beam splitters (BSs) on wavelength to cause legitimate users Alice and Bob to underestimate their excess noise so that Eve can steal more secret keys without being detected. Recently, the wavelength attack on Gaussian-modulated continuous-variable quantum key distribution (CV-QKD) has been researched in both fiber and atmospheric channels. However, the wavelength attack may also pose a threat to the case of ocean turbulent channels, which are vital for the secure communication of both ocean sensor networks and submarines. In this work, we propose two wavelength attack schemes on underwater discrete modulated (DM) CV-QKD protocol, which is effective for the case with and without local oscillator (LO) intensity monitor, respectively. In terms of the transmittance properties of the fused biconical taper (FBT) BS, two sets of wavelengths are determined for Eve’s pulse manipulation, which are all located in the so-called blue–green band. The derived successful criterion shows that both attack schemes can control the estimated excess noise of Alice and Bob close to zero by selecting the corresponding condition parameters based on channel transmittance. Additionally, our numerical analysis shows that Eve can steal more bits when the wavelength attack controls the value of the estimated excess noise closer to zero.

## 1. Introduction

One of the most famous applications of quantum mechanics is quantum key distribution (QKD), which can protect distant communication with unconditional security [1]. Generally, QKD has two main categories: the discrete-variable (DV) version [2,3,4] and the continuous-variable (CV) version [5,6,7]. In detail, DV-QKD encodes the information on the polarization of a photon and uses a single-photon detector for receiver detection, while CV-QKD encodes the information on the quadrature of the optical field and uses coherent detection. Compared with the DV version, CV-QKD is a younger category but has the advantages of lower cost detection and higher compatibility with the current optical communication system [8,9,10].

To date, many CV-QKD protocols have been proposed. By using different quantum states, CV-QKD has a coherent-state protocol and a squeezed-state protocol [5,11]. By using various modulation formats, CV-QKD has a Gaussian-modulated protocol and a discrete modulated (DM) protocol [5,12]. Among these protocols, research on the Gaussian-modulated coherent-state (GMCS) protocol, whose security has been proven under individual attacks [13], collective attacks [14], and coherent attacks [15], is the most advanced. However, unbounded Gaussian modulation can only be implemented to a certain degree in practice. Moreover, for the GMCS protocol, it is hard to achieve highly efficient error correction at a low signal-to-noise ratio (SNR). Therefore, the DM protocol was first proposed in 2009 to improve error correction efficiency in low-SNR cases [16]. In detail, the discrete signals are loaded symmetrically on the quadrature phase, similar to phase-shift keying (PSK) in digital communication systems.

When we develop a security proof, no matter the GMCS protocol or DM protocol, we assume that all devices follow their ideal mathematical models. In the real world, however, the device may deviate from the ideal model and introduce practical security loopholes because of Eve’s manipulation, such as saturation attacks [17], Trojan-horse attacks [18], and so on. In Ref. [19], the authors proposed a local oscillator (LO) calibration attack, where Eve manipulates the linear relationship between the variance in the measurement and the intensity of the LO by changing the shape of the LO signal. In detail, the calibration attack makes the legitimate users overestimate the shot noise so that they underestimate the channel excess noise. Therefore, Eve can perform an intercept–resend attack without being detected. To defend systems against this attack, the authors suggest randomly attenuating the signal intensity to monitor the shot noise [19]. However, this defense method was demonstrated not to hold when the wavelength attack was proposed [20]. The wavelength attack uses the imperfection of the beam splitter (BS) to make the users overestimate the shot noise again and even perform shot noise monitoring. Recently, the wavelength attack on CV-QKD in a free-space atmospheric channel, as well as in the fiber case, was analyzed and proven to be effective [21]. As discussed in many papers [22,23,24,25], the atmospheric channel is an important part of building a global quantum network, especially at present, when quantum relay technology is not yet mature [26].

The free-space seawater channel, like the atmospheric channel, is also an important part of the global quantum network, as it can be used for ocean exploitation and modern communication [27]. Even if the communication capability of the seawater quantum channel has been discussed in Ref. [28], seawater research lags behind that of the atmosphere. In fact, the seawater channel is a promising channel for quantum tasks, as free-space water is a uniform isotropic medium which does not lead to massive polarization rotation or depolarization of single photons [29]. Motivated by the previous idea, in this work, we explore the wavelength attack on CV-QKD in a seawater channel. We consider the four-state DM protocol with homodyne detection, while the central wavelength of Alice and Bob is located in the blue–green band for lower attenuation [30]. In terms of the determined central wavelength and the transmittance properties of fused biconical taper (FBT) BSs, we determine two sets of wavelengths for Eve, which are randomly selected by Eve when she manipulates the pulse. Numerical analysis shows that the wavelength attack can steal the secret key without being detected by manipulating the estimated excess noise of Alice and Bob.

The paper is organized as follows: In Section 2, we show the influences of horizontal seawater links, including extinction losses and ocean turbulence. In Section 3, we review the principle of the four-state protocol and FBT BSs and then propose two wavelength attack schemes. In Section 4, we derive the successful criteria of both attack schemes. In Section 5, we show the impact of wavelength attack on the security of the four-state protocol. Finally, Section 6 draws a conclusion.

## 2. Seawater Channel

In this section, we present a brief introduction to transmittance in seawater channels. The channel model used in our manuscript has been discussed in our previous works [28]. Specifically, this model considers the effects caused by extinction and turbulence in a horizontal seawater link.

### 2.1. Extinction Losses

The extinction-induced losses are caused by the absorption and scattering of both soluble and insoluble impurities, such as chlorophyll, inorganic salts, sediment particles, microorganisms, etc. Specifically, absorption causes energy loss while scattering leads to the divergence of the laser beam [31,32], and they are quantified by absorption coefficient $a\left(\lambda \right)$ and scattering coefficient $b\left(\lambda \right)$, respectively. Here, λ denotes the wavelength of quantum light, while the details of $a\left(\lambda \right)$ and $b\left(\lambda \right)$ are shown in Appendix A of Ref. [28]. In detail, $a\left(\lambda \right)$ and $b\left(\lambda \right)$ are related to both the ocean type and the submarine depth *d*. The total extinction coefficient $\beta \left(\lambda \right)$ is defined as the sum of $a\left(\lambda \right)$ and $b\left(\lambda \right)$. Finally, extinction-induced transmittance is characterized by the Lambert–Beer law given by [28].

(1) $$\eta_{\mathrm{ext}}=e^{-\beta (\lambda )L},$$

where *L* is the transmission distance. For less attenuation, we use a 532 nm laser in the blue–green band in the following. In addition, the ocean type of our manuscript is set to S6 as an example.

### 2.2. Ocean Turbulence

In general, ocean turbulence is caused by the combined effect of two fluctuating scalars: temperature and salinity. In our manuscript, these scalars can be characterized by the classical Kolmogorov power spectrum given by [33].

(2) $$\Phi \left(\kappa \right)=A\omega \xi^{-\frac{1}{3}}\kappa^{-\frac{11}{3}},$$

where A is the order of unity, ω is related to the dissipation rate of temperature or salinity variance, ξ is the kinetic energy dissipation rate, and κ is the spatial frequency. In terms of this power spectrum, the elliptical model of free-space quantum light can be used for analyzing the beam evolution of ocean turbulent channels. Finally, transmittance in a seawater channel with an initial beam radius $w_{0}$ can be estimated as

(3) $$\eta_{\mathrm{ch}}=\eta_{\mathrm{ext}}\eta_{0}\exp\left\{-{\left[\frac{r/a}{Q(\frac{2}{w_{\mathrm{eff}}\left(\phi -\varphi \right)})}\right]}^{Y\left(\frac{2}{w_{\mathrm{eff}}\left(\phi -\varphi \right)}\right)}\right\},$$

where $\eta_{0}$ is the transmittance without either extinction and beam wandering effects, *r* is the beam-centroid vector, *a* is the receiver telescope radius, $w_{\mathrm{eff}}\left(\cdot \right)$ is the effective spot-radius with deformation effects, ϕ is the beam–ellipse orientation angle, φ denotes the angle between vector *r* and the *x*-axis, and Q(·) and Y(·) are scale and shape functions, respectively. The details of the above parameters are shown in Appendix B of Ref. [28]. In our manuscript, the value of the above parameters are *a* = 0.25 m, $w_{0}$ = 80 mm, $\omega =10^{-11}$, and $\xi =10^{-3}$.

## 3. Seawater Wavelength Attack on Discrete Modulated CV-QKD

In this section, we have a quick review of the four-state DM protocol and the principle of FBT BSs. Then, two wavelength attack schemes in seawater channels are proposed.

### 3.1. The Four-State Protocol

The four-state protocol with homodyne detection will be used in the following, and its steps can be described as follows:

(1) *State preparation and transmission:* For the *j*-th round, Alice randomly prepares one of the quantum states from $|\phi_{j}\rangle \in \left\{|\alpha e^{i(2k-1)\pi /4}\rangle :k\in 1,2,3,4\right\}$ and sends it to Bob via a thermal-loss channel, which is characterized by transmittance $\eta_{\mathrm{ch}}$ and excess noise ε. Here, α is the amplitude of the quantum state, and the states $|\alpha e^{i(\pi /4)}\rangle$, $|\alpha e^{i(3\pi /4)}\rangle$, $|\alpha e^{i(4\pi /4)}\rangle$, and $|\alpha e^{i(7\pi /4)}\rangle$ correspond to the data 00, 10, 11, and 01, respectively. Along with the quantum state, Alice also prepares and sends a strong LO by multiplexing technology for homodyne detection on Bob’s side.
(2) *Measurement:* With the help of the multiplexed LO, Bob performs homodyne detection on the *q* or *p* quadrature of the arriving quantum state for the raw key. In detail, Bob generates a uniform random number $b_{j}\in \left\{1,2\right\}$ so that $b_{j}=0$ ($b_{j}=1$ ) measures *q* (*p*).
(3) *Parameter estimation:* To obtain a practical secret key rate, Alice and Bob choose a part of the data for parameter estimation. In detail, Alice publishes part of the quantum states she sends, and Bob publishes the corresponding measurement results. Based on the public information, both parties can estimate the practical secret key rate. If this secret key rate is below zero, both parties abort the protocol; otherwise, they proceed to the data post-processing.
(4) *Data post-processing:* General data post-processing has two steps: error correction and privacy amplification. Error correction is to correct the keys that are inconsistent between the two parties. Private amplification reduces the amount of information to which Eve has access to with the secret key. After the appropriate private amplification, Alice and Bob generate the final secret key.

Note that the above description is based on the prepare-and-measure scheme, which is widely used in experiments. For the security analysis, one usually uses an equivalent entanglement-based (EB) scheme, as shown in Section 5.

### 3.2. The Principle of Beam Splitters

Generally, the BS used in the CV-QKD system is an FBT BS, which combines the ends of two bare fibers in a high-temperature environment to form a biconical waveguide structure. The splitting ratio of this BS is related to the wavelength of the input, which can be expressed as [34]

(4) $$T\left(\lambda \right)=F^{2}\sin^{2}\left(\frac{c\lambda^{2.5}w}{F}\right),$$

where $F^{2}$ denotes the fraction of power coupled, $c\lambda^{2.5}$ represents the coupling coefficient of the FBT BS, and *w* is the width of the heat source. As discussed in Section 2.1, our manuscript uses a central wavelength $\lambda_{0}$ = 532 nm for less attenuation. Therefore, we have $T\left(\lambda_{0}\right)=\sin^{2}\left(c{\lambda_{0}}^{2.5}w\right)=0.5$. Here, we set *F* = 1 for simplicity. Based on Equation (4), we find that only when the input is located at the central wavelength, the splitting ratio of the BS is 50:50. In other words, the splitting ratio is no longer balanced when the input’s wavelength deviates from the center wavelength. In what follows, we will discuss how Eve uses the wavelength dependence of such BS for the so-called wavelength attack.

### 3.3. Wavelength Attack Scheme

**Figure 1:**
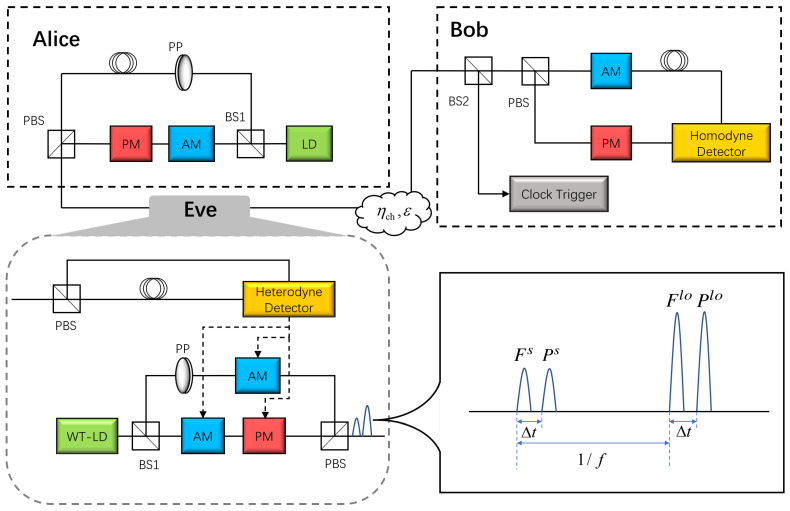
Seawater wavelength attack scheme using homodyne detector. LD, laser diode; WT-LD, wavelength-tunable laser diode; PP, polarizing prism; BS1, 1:99 beam splitter; BS2, 10:90 beam splitter; PBS, polarization beam splitter; AM, amplitude modulator; PM, phase modulator; Δt, a very small time interval; *f*, repetition rate.

Figure 1 shows the general wavelength attack scheme. To implement the wavelength attack, Eve first intercepts and measures both the *q* and *p* quadrature of Alice’s quantum states $|\phi_{j}\rangle$ by heterodyne detection. Then, Eve prepares and sends two groups of pulses at the same time to Bob: $\left\{F^{s},F^{lo}\right\}$ and $\left\{P^{s},P^{lo}\right\}$. In detail, the wavelength of $\left\{F^{s},F^{lo}\right\}$ is the same as Alice, while the wavelength of $\left\{P^{s},P^{lo}\right\}$ is changed by Eve. In the first group, the signal pulse $F^{s}$ is modulated according to Eve’s measurement results $\left\{q_{E},p_{E}\right\}$, and the LO pulse $F^{lo}$ is manipulated by Eve in terms of Bob’s monitoring method. In the second group, the wavelengths of $\left\{P^{s},P^{lo}\right\}$ (i.e., $\lambda^{s}$ and $\lambda^{lo}$) are randomly selected from two sets of wavelengths with equal probability. In terms of the central wavelength $\lambda_{0}$, the two sets of wavelengths in our manuscript are shown in Table 1, which also shows the corresponding transmittance, i.e., $T^{s}$ and $T^{lo}$ [35].

On the receiver side, the differential current measured by the homodyne detector comes from the signal photocurrent $i^{s}$ and LO photocurrent $i^{lo}$, whose intensities are $I^{s}$ and $I^{lo}$, respectively. In general, Bob will attenuate the quantum signal with randomly selected coefficient $r_{i}$ (*i* = 1, 2), which equals 0 or 1, to resist the LO calibration attack [19]. In detail, when $r_{1}\approx 0$, the differential current is primarily contributed by $i^{lo}$. If $T^{lo}$ = 0.5, which correspond to Bob measuring $\left\{F^{s},F^{lo}\right\}$, only the differential current remains.

(5) $$i^{sn}=\sqrt{\eta^{lo}I^{lo}}\left(\sqrt{\eta^{lo}}\delta {\hat{X}}_{\phi }+\sqrt{1-\eta^{lo}}\frac{{\hat{X}}_{v2}-{\hat{X}}_{v1}}{\sqrt{2}}\right),$$

where $\delta {\hat{X}}_{\phi }$, ${\hat{X}}_{v1}$, and ${\hat{X}}_{v2}$ are irrelevant vacuum states and $\eta^{lo}$ is the detection efficiency when the wavelength is $\lambda_{lo}$. Note that the variance in Equation (5) is used as the normalized shot noise unit given by

(6) $$N_{0}=\eta^{lo}I^{lo}=\eta^{lo}\alpha_{\mathrm{lo}}^{2},$$

where $\alpha_{\mathrm{lo}}=\sqrt{I_{lo}}$ is the amplitude of the LO. If $T^{lo}\neq 0.5$, which means that Bob measures $\left\{P^{s},P^{lo}\right\}$, the differential current (without shot noise part) is approximately equal to [20]

(7) $$D^{lo}=\left(2T^{lo}-1\right)\eta^{lo}I^{lo}.$$

When $r_{2}\approx 1$, the differential current is contributed by both $i^{s}$ and $i^{lo}$. If Bob measures $\left\{F^{s},F^{lo}\right\}$, we have $T^{s}=T^{lo}=0.5$ and obtain the $q_{E}$ or $p_{E}$ quadrature. If Bob measures $\left\{P^{s},P^{lo}\right\}$, we have $T^{s}\approx T^{lo}\neq 0.5$ (see Table 1), and the differential current (without shot noise part) is approximately equal to

(8) $$D^{s}=\left(1-2T^{s}\right)\eta^{s}I^{s},$$

where $\eta^{s}$ is the detection efficiency when wavelength is $\lambda_{s}$.

**Figure 2:**
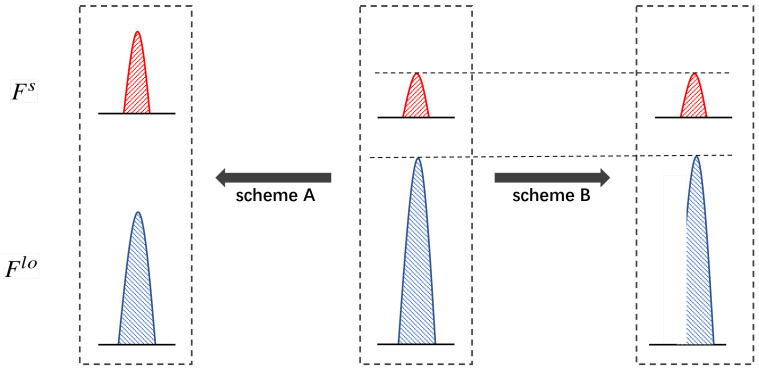
Differences in Eve’s manipulation between attack scheme A and attack scheme B. Red and blue colors represent signal pulse and LO pulse, respectively.

As mentioned above, the LO pulse $F^{lo}$ is manipulated by Eve in terms of Bob’s monitoring method. Next, we discuss two attack schemes where Bob is without and with LO intensity monitoring ability, i.e., attack scheme A and attack scheme B, respectively. Figure 2 shows the differences in Eve’s manipulation between attack scheme A and attack scheme B. In attack scheme A, both the amplitudes of the signal and the LO are manipulated, while attack scheme B only manipulates the wave shape of the LO.

**Attack scheme A**: If Bob does not measure the intensity of the LO, Eve performs the intercept–resend attack along with the wavelength attack. In this case, Bob uses the shot noise unit obtained before key distribution to normalize the measurement result. This scheme has two parts, as follows.

*Part 1*: Eve performs the intercept–resend attack and obtains the heterodyne detection results $\left\{q_{E},p_{E}\right\}$. Then, Eve sends $\left\{F^{s},F^{lo}\right\}$ according to $\left\{q_{E},p_{E}\right\}$ and keeps their polarization unchanged. The amplitude of $F^{s}$ is set to $\sqrt{N\eta_{\mathrm{ch}}}\left(q_{E}+ip_{E}\right)/2$, where *N* is larger than 1. For the amplitude of $F^{lo}$, Eve changes it from $\alpha_{\mathrm{lo}}$ to $\alpha_{\mathrm{lo}}/\sqrt{N}$.

*Part 2*: Eve sends $\left\{P^{s},P^{lo}\right\}$ at the same time as $\left\{F^{s},F^{lo}\right\}$, and the wavelengths of $\left\{P^{s},P^{lo}\right\}$ are chosen randomly from Table 1.

In Part 1, Eve reduces the amplitude of $F_{lo}$, which will decrease the shot noise unit. In this case, Bob can detect Eve’s attack by shot noise monitoring if Eve has no other steps. Therefore, Eve needs to add $\left\{P^{s},P^{lo}\right\}$ in Part 2 to compensate the total shot noise unit to a normal level, i.e., $N_{0}$. In detail, the variance in these pulses’ differential current is considered the added shot noise. However, if Eve only uses one set of wavelengths, such as Set 1, the variance in the corresponding differential current $D_{1}^{lo}$ will equal zero. This is the reason why we use two sets of wavelengths with random selection. In addition to the variance, the mean value of $D^{lo}$ should be zero because a normal shot noise quadrature is considered a random variable with zero mean value. Therefore, we set $T_{1}^{lo}\approx 1-T_{2}^{lo}$ to make $D_{1}^{lo}=-D_{2}^{lo}$ so that $\langle D^{lo}\rangle =\langle D_{1}^{lo}\rangle +\langle D_{2}^{lo}\rangle =0$. Finally, the mean value of $D^{s}$ should also be zero, so that we set $D_{1}^{s}=-D_{2}^{s}$. In total, we can make $D_{1}^{s}=-D_{1}^{lo}=-D_{2}^{s}=D_{2}^{lo}\equiv D$ for simple.

**Attack scheme B**: If Bob measures the intensity of the LO, Eve cannot reduce the amplitude of $F^{lo}$ anymore. In this case, Eve combines the LO calibration attack and wavelength attack. Note that attack scheme B also uses $D_{1}^{s}=-D_{1}^{lo}=-D_{2}^{s}=D_{2}^{lo}\equiv D$ to meet the requirements mentioned above. The two parts of this scheme are as follows.

*Part 1*: Eve performs the intercept–resend attack and sends $F^{s}$ according to $\left\{q_{E},p_{E}\right\}$, whose amplitude is $\sqrt{\eta_{\mathrm{ch}}}\left(X_{E}+iP_{E}\right)/2$. Then, Eve performs an LO calibration attack, which changes the shape of the LO to delay Bob’s detector response time. This change makes Bob overestimate the shot noise unit with the correct LO intensity.

*Part 2*: Same as Part 2 in attack scheme A.

## 4. The Successful Criterion of the Wavelength Attack

Considering the random coefficient $r_{i}$, the variance in Bob’s homodyne detection data under the linear channel assumption is

(9) $${\langle {\hat{y}}^{2}\rangle }_{i}=r_{i}\eta \eta_{\mathrm{ch}}\left(V_{A}+\varepsilon \right)N_{0}+N_{0}+v_{\mathrm{el}}N_{0},$$

where $V_{A}$ is the modulation variance and $v_{\mathrm{el}}$ is the electronic noise. Then, Bob can estimate the shot noise unit and the excess noise as

(10) $${\hat{N}}_{0}=\left[\frac{r_{2}{\langle {\hat{y}}^{2}\rangle }_{1}-r_{1}{\langle {\hat{y}}^{2}\rangle }_{2}}{r_{2}-r_{1}}\right]/\left(1+v_{\mathrm{el}}\right),$$

(11) $$\tilde{\varepsilon }=\left[\frac{{\langle {\hat{y}}^{2}\rangle }_{2}-{\langle {\hat{y}}^{2}\rangle }_{1}}{\left(r_{2}-r_{1}\right)\eta \eta_{\mathrm{ch}}}-V_{A}{\hat{N}}_{0}\right]/{\hat{N}}_{0}.$$

To ensure the success of the attack without being detected, the following two conditions must be met: ${\hat{N}}_{0}=N_{0}$ and $\tilde{\varepsilon }\leq \varepsilon$. Next, we derive the specific success criteria of the two attack schemes. In the following, the values of the parameters are $V_{A}=0.3$ [36], ε=0.1, $v_{\mathrm{el}}=0.01$, $\eta_{1}^{s}=\eta_{2}^{s}=\eta_{1}^{lo}=\eta_{2}^{lo}=\eta =0.5$, $I^{lo}=1\times 10^{8}$, $r_{1}=0.001$, $r_{2}=1$, and $N_{0}=\eta I^{lo}=5\times 10^{7}$.

### 4.1. Attack Scheme A

The differential current measured by the homodyne detector is the sum of the currents from two parts, which can be expressed as

(12) $$\hat{\delta }i_{\mathrm{tot},i}=\hat{\delta }i_{\mathrm{part}1,i}+\hat{\delta }i_{\mathrm{part}2,i},$$

where i={1,2} corresponds to the coefficients $\left\{r_{1},r_{2}\right\}$, respectively. In detail, the variance in $\hat{\delta }i_{\mathrm{part}1,i}$ is given by

(13) $$\begin{array}{cc} V_{\mathrm{part}1,i}^{A} & =\eta \frac{\alpha_{LO}^{2}}{N}\left[r_{i}\eta \eta_{\mathrm{ch}}N\left(V_{A}+2\right)+1\right]+r_{i}\eta \eta_{\mathrm{ch}}\varepsilon N_{0}+v_{el}N_{0}\\ \; & =r_{i}\eta \eta_{\mathrm{ch}}\left(V_{A}+2+\varepsilon \right)N_{0}+\frac{N_{0}}{N}+v_{\mathrm{el}}N_{0}. \end{array}$$

Then, the variance in $\hat{\delta }i_{\mathrm{part}2,i}$ can be expressed as

(14) $$V_{\mathrm{part}2,i}^{A}={\left(1-r_{i}\right)}^{2}D^{2}+\eta \langle I_{j}^{lo}\rangle +\eta r_{i}^{2}\langle I_{j}^{s}\rangle ,$$

where $\langle I_{j}^{lo}\rangle$ and $\langle I_{j}^{s}\rangle$ denote the mean values of $I_{j}^{lo}$ and $I_{j}^{s}$ in the seawater channel, respectively. Since we randomly select the coefficient $r_{i}$, $\langle I_{j}^{lo}\rangle$ and $\langle I_{j}^{s}\rangle$ are given by

(15) $$\langle I_{j}^{lo}\rangle =\frac{I_{1}^{lo}}{2}+\frac{I_{2}^{lo}}{2}=\frac{D_{1}^{lo}}{2\eta \left(2T_{1}^{lo}-1\right)}+\frac{D_{2}^{lo}}{2\eta \left(2T_{2}^{lo}-1\right)}=45.854D,$$

(16) $$\langle I_{j}^{s}\rangle =\frac{I_{1}^{s}}{2}+\frac{I_{2}^{s}}{2}=\frac{D_{1}^{s}}{2\eta \left(1-2T_{1}^{s}\right)}+\frac{D_{2}^{s}}{2\eta \left(1-2T_{2}^{s}\right)}=45.170D.$$

Therefore, Equation (14) can be rewritten as

(17) $$V_{\mathrm{part}2,i}^{A}={\left(1-r_{i}\right)}^{2}D^{2}+\left(22.927+22.585r_{i}^{2}\right)D.$$

Thus, the variance in the differential current can be expressed as

(18) $$\begin{array}{cc} {\langle {\hat{y}}^{2}\rangle }_{i}^{A} & =V_{\mathrm{part}1,m}^{A}+V_{\mathrm{part}2,m}^{A}\\ \; & =r_{m}\eta \eta_{\mathrm{ch}}\left(V_{A}+2+\varepsilon \right)N_{0}+\frac{N_{0}}{N}+v_{\mathrm{el}}N_{0}+{\left(1-r_{m}\right)}^{2}D^{2}+\left(22.927+22.585r_{m}^{2}\right)D. \end{array}$$

Finally, Bob estimates the shot noise unit and excess noise by Equations (10) and (11), which can be expressed as

(19) $$\begin{array}{cc} {\hat{N}}_{0} & =\frac{\left(\frac{1}{N}+v_{\mathrm{el}}\right)N_{0}+\left(1-r_{1}r_{2}\right)D^{2}+\left(22.927-22.585r_{1}r_{2}\right)D}{1+v_{\mathrm{el}}},\\ \tilde{\varepsilon } & =\left[\left(2+\varepsilon \right)\frac{N_{0}}{{\hat{N}}_{0}}+V_{A}\left(\frac{N_{0}}{{\hat{N}}_{0}}-1\right)+\frac{\left(r_{1}+r_{2}-2\right)D^{2}}{\eta \eta_{\mathrm{ch}}{\hat{N}}_{0}}+\frac{22.585\left(r_{1}+r_{2}\right)D}{\eta \eta_{\mathrm{ch}}{\hat{N}}_{0}}\right]. \end{array}$$

To make the wavelength attack successful, the parameters *N* and *D* should satisfy

(20) $$N=-\frac{N_{0}}{\left(1-r_{1}r_{2}\right)D^{2}+\left(22.927-22.585r_{1}r_{2}\right)D-N_{0}},$$

(21) $$-\left(2+\varepsilon \right)\eta \eta_{\mathrm{ch}}{\hat{N}}_{0}<\left(r_{1}+r_{2}-2\right)D^{2}+22.585\left(r_{1}+r_{2}\right)D\leq -2\eta \eta_{\mathrm{ch}}{\hat{N}}_{0}.$$

We find that these two formulas are not related to $V_{A}$, which implies that Eve can perform the attack without knowing the modulation variance.

### 4.2. Attack Scheme B

In this scheme, since Eve performs the LO calibration attack, the shot noise unit is $\gamma N_{0}$ (γ < 1). Then, the variance in $\hat{\delta }i_{\mathrm{part}1,m}$ is given by

(22) $$V_{\mathrm{part}1,m}^{B}=\gamma \left[r_{m}\eta \eta_{\mathrm{ch}}^{\prime }\left(V_{A}+2+\varepsilon \right)+1\right]N_{0}+v_{\mathrm{el}}N_{0},$$

where $\eta_{\mathrm{ch}}^{\prime }=\eta_{\mathrm{ch}}/\gamma$ can be considered the virtual channel transmittance simulated by Eve. Next, the expression of $V_{\mathrm{part}2,m}^{B}$ is the same as that in attack scheme A. Therefore, the variance in the differential current is

(23) $$\begin{array}{cc} {\langle {\hat{y}}^{2}\rangle }_{i}^{B} & =V_{\mathrm{part}1,m}^{B}+V_{\mathrm{part}2,m}^{B}\\ \; & =\gamma \left[r_{m}\eta \eta_{\mathrm{ch}}^{\prime }\left(V_{A}+2+\varepsilon \right)+1\right]N_{0}+v_{\mathrm{el}}N_{0}+{\left(1-r_{m}\right)}^{2}D^{2}+\left(22.927+22.585r_{m}^{2}\right)D. \end{array}$$

Based on Equation (10), Bob estimates the variance in the shot noise given by

(24) $${\hat{N}}_{0}=\frac{\left(\gamma +v_{\mathrm{el}}\right)N_{0}+\left(1-r_{1}r_{2}\right)D^{2}+\left(22.927-22.585r_{1}r_{2}\right)D}{1+v_{\mathrm{el}}};$$

thus, γ should satisfy

(25) $$\gamma =1-\frac{\left(1-r_{1}r_{2}\right)D^{2}+\left(22.927-22.585r_{1}r_{2}\right)D}{N_{0}}.$$

Based on Equation (11), we find that the estimation of $\tilde{\varepsilon }$ is the same as that of attack scheme A. We find that the above formulas are still not related to $V_{A}$.

## 5. Simulation

**Figure 3:**
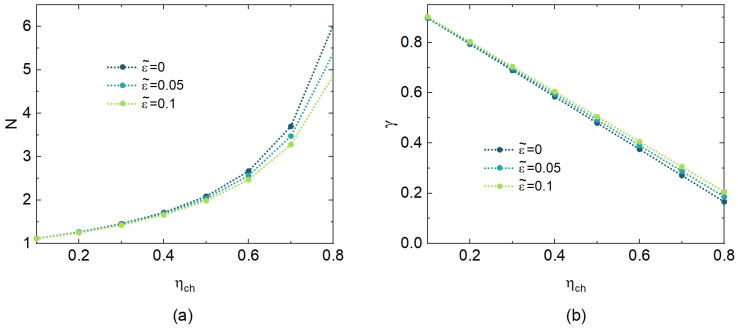
(**a**) *N* of attack scheme A vs. transmittance for various $\hat{\varepsilon }$. (**b**) γ of attack scheme B vs. transmittance for various $\hat{\varepsilon }$.

In this section, we first show the effectiveness of our two attack schemes. Then, the secret key rate and Holevo bound of the four-state protocol are discussed. The simulation parameters are the same as in Section 4. As discussed in Section 4, the condition parameters in attack scheme A and attack scheme B are *N* and γ, respectively. Figure 3 shows the relationship of these two parameters with various $\tilde{\varepsilon }$ and transmittance $\eta_{\mathrm{ch}}$. Here, the green lines represent the case $\tilde{\varepsilon }=\varepsilon =0.1$, which means Eve cannot obtain more information by the wavelength attack. We find that both schemes can control $\tilde{\varepsilon }$ close to zero. To a target $\tilde{\varepsilon }$, the corresponding *N* of attack scheme A increases as the transmittance increases, as shown in Figure 3a. Moreover, for each transmittance value, Eve needs to use a lager *N* for a lower $\tilde{\varepsilon }$. In contrast, Figure 3b shows that the required value of γ decreases as the transmittance grows, while a lower γ obtains a lower $\tilde{\varepsilon }$ for each transmittance.

As described in Section 3.1, in parameter estimation, Bob calculates the practical secret key rate according to the estimated channel parameters. Here, we use the equivalent EB scheme instead of the prepare-and-measure scheme for security analysis. In the equivalent EB scheme, Alice generates a two-mode entangled state, which can be characterized by its covariance matrix given by [36].

(26) $$\gamma_{AB}=\left[\begin{array}{cc} VI_{2} & Z_{4}\sigma_{z}\\ Z_{4}\sigma_{z} & VI_{2} \end{array}\right],$$

where $I_{2}=\mathrm{diag}\left(1,1\right)$, $\sigma_{z}=\mathrm{diag}\left(1,1\right)$, $V=V_{A}+1$, and

(27) $$Z_{4}=2\alpha^{2}\left(\frac{p_{0}^{3/2}}{p_{1}^{1/2}}+\frac{p_{1}^{3/2}}{p_{2}^{1/2}}+\frac{p_{2}^{3/2}}{p_{3}^{1/2}}+\frac{p_{3}^{3/2}}{p_{0}^{1/2}}\right),$$

with

(28) $$p_{0,2}=\frac{1}{2}e^{-\alpha^{2}}\left[\cosh\left(\alpha^{2}\right)\pm \cos\left(\alpha^{2}\right)\right],$$

(29) $$p_{1,3}=\frac{1}{2}e^{-\alpha^{2}}\left[\sinh\left(\alpha^{2}\right)\pm \sin\left(\alpha^{2}\right)\right].$$

Then, mode *A* of the entangled state stays on Alice’s side for heterodyne measurement, while mode *B* is transferred to Bob by the quantum channel. When mode *B* reaches Bob, the covariance matrix of the arriving entangled states can be expressed as [36]

(30) $$\gamma_{AB}=\left[\begin{array}{cc} VI_{2} & \langle \sqrt{\eta_{\mathrm{ch}}}\rangle Z_{4}\sigma_{z}\\ \langle \sqrt{\eta_{\mathrm{ch}}}\rangle Z_{4}\sigma_{z} & \left[\langle \eta_{\mathrm{ch}}\rangle \left(V-1\right)+1+\langle \eta_{\mathrm{ch}}\rangle \tilde{\varepsilon }\right]I_{2} \end{array}\right].$$

In the asymptotic case with reverse reconciliation, the secret key rate can be expressed as

(31) $$K\left(\langle \eta_{\mathrm{ch}}\rangle ,\tilde{\varepsilon }\right)=\beta_{0}I_{AB}\left(\langle \eta_{\mathrm{ch}}\rangle ,\tilde{\varepsilon }\right)-\chi_{BE}\left(\langle \eta_{\mathrm{ch}}\rangle ,\tilde{\varepsilon }\right),$$

where 〈·〉 means the mean value, $I_{AB}$ is the classic mutual information between Alice and Bob, $\chi_{BE}$ is the Holevo bound, and β is the reconciliation efficiency (see Appendix A for details).

**Figure 4:**
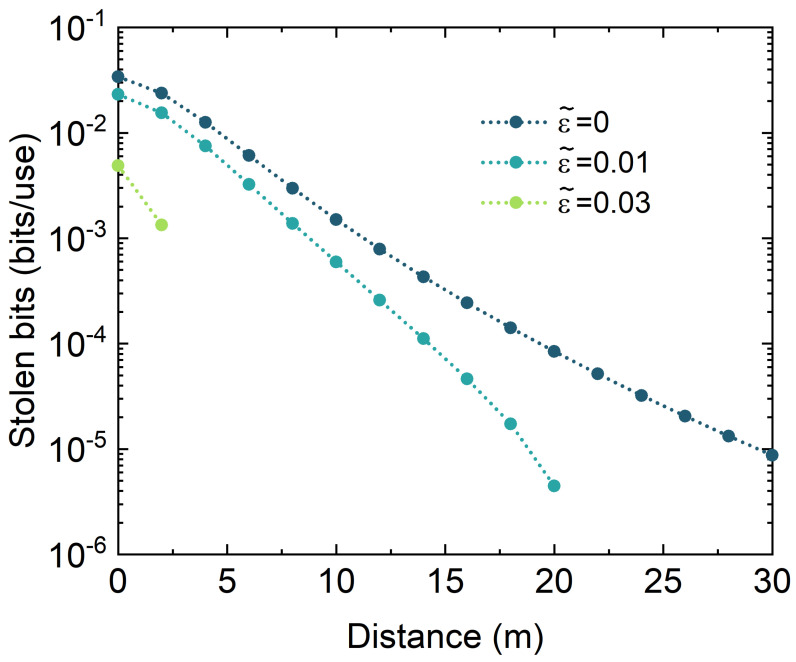
The stolen bits in the S6 ocean for various estimated excess noise values $\tilde{\varepsilon }$. The submarine depth is set to *d* = 200 m.

**Figure 5:**
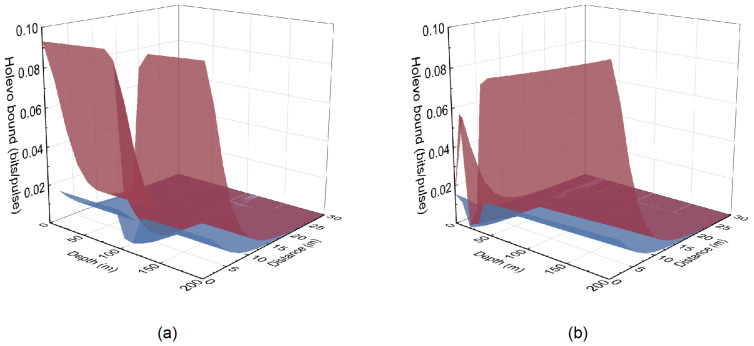
The relationship of the real Holevo bound $\chi_{BE}\left(\langle \eta_{\mathrm{ch}}\rangle ,\varepsilon \right)$ (red surface) and the estimated Holevo bound $\chi_{BE}\left(\langle \eta_{\mathrm{ch}}\rangle ,\tilde{\varepsilon }\right)$ (blue surface) with various submarine depths and transmission distances. The estimated excess noise is set to $\tilde{\varepsilon }$ = 0. (**a**) S1 seawater. (**b**) S6 seawater.

Figure 4 shows the relationship between the stolen bits, i.e., $K\left(\langle \eta_{\mathrm{ch}}\rangle ,\tilde{\varepsilon }\right)-K\left(\langle \eta_{\mathrm{ch}}\rangle ,\varepsilon \right)$, and transmission distance *L* when using the four-state protocol with submarine depth *d* = 200 m. Here, we use reverse reconciliation with reconciliation efficiency $\beta_{0}$ = 0.95, while the real excess noise is ε = 0.1. We find that Alice and Bob cannot generate a secret key when ε = 0.1. However, after the wavelength attack, Alice and Bob use the estimated excess noise $\tilde{\varepsilon }$ for the estimation of the secret key rate. In this case, the users will believe the keys are secure when the estimation result is above zero. For example, when Eve reduces the estimated excess noise to zero, the users will believe that the secure transmission distance is above 30 m. In fact, the real secure transmission distance is zero so that Eve can obtain these secret keys without being detected. In addition, the estimated secret key rate increases when $\tilde{\varepsilon }$ decreases. For example, the estimated secure transmission distance increases from 2 m to above 30 m when $\tilde{\varepsilon }$ decreases from 0.03 to 0. Figure 5 shows the relationship of the real Holevo bound $\chi_{BE}\left(\langle \eta_{\mathrm{ch}}\rangle ,\varepsilon \right)$ and the estimated Holevo bound $\chi_{BE}\left(\langle \eta_{\mathrm{ch}}\rangle ,\tilde{\varepsilon }\right)$ under different seawater types with various submarine depth *d* and transmission distance *L*. Here, the estimated Holevo bound is calculated with an estimated excess noise $\tilde{\varepsilon }$ = 0. We find that there is a big difference in the performance of the two seawater types.

## 6. Conclusions and Discussion

In this paper, we have proposed two wavelength attack schemes for the underwater four-state protocol with and without LO intensity monitoring. Transmittance in underwater channels is affected by both extinction and ocean turbulence, so it fluctuates randomly over time. Different from both the fiber and atmosphere cases, the communication wavelength of underwater channels is not 1550 nm but is located in the so-called blue–green band. To meet this change, we have proposed two sets of wavelengths for Eve’s pulse manipulation based on the transmittance properties of FBT BSs. We have calculated the successful criteria of both attack schemes and found that Eve can manipulate the estimated excess noise of Alice and Bob close to zero by slightly changing the corresponding condition parameters, i.e., *N* or γ. Numerical analysis shows that the secure transmission distance is overestimated by Alice and Bob when Eve performs the wavelength attack.

To avoid the wavelength attack, a direct idea is that one can design and use a wavelength-independent BS instead of a wavelength-dependent one. In addition, narrow wavelength filtering on Bob’s side can also avoid the wavelength attack. Note that wavelength filtering needs LO intensity monitoring to work together. This is because practical wavelength filtering has an upper limit of attenuation for any specific wavelength so that Eve can beat it by increasing the pulse intensity. Moreover, Ref. [20] has proposed a method by using a third attenuation ratio in the shot noise monitoring module. In this method, Alice and Bob ensure they avoid a wavelength attack when the polynomial function of the total noise-to-attenuation ratio is almost linear. To avoid increasing the complexity and decreasing the final secret key rate in the above method, Ref. [37] has proposed another data post-processing method via peak–valley seeking and Gaussian postselection.

## Figures and Tables

**Table 1:** Two sets of wavelengths with corresponding transmittance.

Set	$\lambda^{s}\left(\mathrm{nm}\right)$	$T^{s}$	$\lambda^{\mathrm{lo}}\left(\mathrm{nm}\right)$	$T^{\mathrm{lo}}$
1	526	0.47805	632	0.47837
2	538	0.52233	637	0.52199

## Data Availability

All data generated or analyzed during this study are included in this article.

## Appendix A

In terms of Equation (30), when using homodyne detection, the mutual information between Alice and Bob can be expressed as

(A1) $$I_{AB}\left(\langle \eta_{\mathrm{ch}}\rangle ,\tilde{\varepsilon }\right)=\frac{1}{2}\log_{2}\frac{1}{1-\frac{{\langle \sqrt{\eta_{\mathrm{ch}}}\rangle }^{2}\left(V-1\right)}{\langle \eta_{\mathrm{ch}}\rangle \left(V+\chi_{\mathrm{tot}}\right)}},$$

where $\chi_{\mathrm{tot}}=\chi_{\mathrm{line}}+\chi_{\mathrm{hom}}/\langle \eta_{\mathrm{ch}}\rangle$ with the total channel-added noise $\chi_{\mathrm{line}}=1/\langle \eta_{\mathrm{ch}}\rangle -1+\tilde{\varepsilon }$ and the detection-added noise $\chi_{\mathrm{hom}}=\left(1-\eta +v_{\mathrm{el}}\right)/\eta$. Then, the estimated Holevo bound $\chi_{BE}\left(\langle \eta_{\mathrm{ch}}\rangle ,\tilde{\varepsilon }\right)$ can be expressed by

(A2) $$\chi_{BE}\left(\langle \eta_{\mathrm{ch}}\rangle ,\tilde{\varepsilon }\right)=\sum_{i=1}^{2}G\left(\frac{\lambda_{i}-1}{2}\right)-\sum_{i=3}^{5}G\left(\frac{\lambda_{i}-1}{2}\right),$$

where $G\left(x\right)=\left(x+1\right)\log_{2}\left(x+1\right)-x\log_{2}x$ and $\lambda_{i}$ represents the symplectic eigenvalues given by

(A3) $$\begin{array}{cc} \; & \lambda_{1,2}^{2}=\frac{1}{2}\left(A\pm \sqrt{A^{2}-4B}\right),\\ \; & \lambda_{3,4}^{2}=\frac{1}{2}\left(C\pm \sqrt{C^{2}-4D}\right),\\ \; & \lambda_{5}=1, \end{array}$$

where

(A4) $$\begin{array}{cc} A & =V^{2}+{\langle \eta_{\mathrm{ch}}\rangle }^{2}{\left(V+\frac{1-\langle \eta_{\mathrm{ch}}\rangle }{\langle \eta_{\mathrm{ch}}\rangle }+\tilde{\varepsilon }\right)}^{2}-2{\langle \sqrt{\eta_{\mathrm{ch}}}\rangle }^{2}Z_{4}^{2},\\ B & ={\left[\langle \eta_{\mathrm{ch}}\rangle V^{2}+\langle \eta_{\mathrm{ch}}\rangle \chi_{\mathrm{line}}V-{\langle \sqrt{\eta_{\mathrm{ch}}}\rangle }^{2}Z_{4}^{2}\right]}^{2},\\ C & =\frac{A\chi_{\mathrm{hom}}+V\sqrt{B}+\langle \eta_{\mathrm{ch}}\rangle \left(V+\chi_{\mathrm{line}}\right)}{\langle \eta_{\mathrm{ch}}\rangle \left(V+\chi_{\mathrm{tot}}\right)},\\ D & =\frac{\sqrt{B}V+B\chi_{\mathrm{hom}}}{\langle \eta_{\mathrm{ch}}\rangle \left(V+\chi_{\mathrm{tot}}\right)}. \end{array}$$
